# Special Issue on “New Advances in Cyclic AMP Signalling”—An Editorial Overview

**DOI:** 10.3390/cells9102274

**Published:** 2020-10-12

**Authors:** Stephen John Yarwood

**Affiliations:** Institute of Biological Chemistry, Biophysics and Bioengineering, School of Engineering and Physical Sciences, Edinburgh Campus, Heriot-Watt University, Edinburgh EH14 4AS, UK; s.yarwood@hw.ac.uk

**Keywords:** cyclic AMP, EPAC, PKA, POPDC, AKAP, adenylate cyclase, cyclic AMP phosphodiesterases, GPCRs

## Abstract

The cyclic nucleotides 3′,5′-adenosine monophosphate (cyclic AMP) signalling system underlies the control of many biological events and disease processes in man. Cyclic AMP is synthesised by adenylate cyclase (AC) enzymes in order to activate effector proteins and it is then degraded by phosphodiesterase (PDE) enzymes. Research in recent years has identified a range of cell-type-specific cyclic AMP effector proteins, including protein kinase A (PKA), exchange factor directly activated by cyclic AMP (EPAC), cyclic AMP responsive ion channels (CICs), and the Popeye domain containing (POPDC) proteins, which participate in different signalling mechanisms. In addition, recent advances have revealed new mechanisms of action for cyclic AMP signalling, including new effectors and new levels of compartmentalization into nanodomains, involving AKAP proteins and targeted adenylate cyclase and phosphodiesterase enzymes. This Special Issue contains 21 papers that highlight advances in our current understanding of the biology of compartmentlised cyclic AMP signalling. This ranges from issues of pathogenesis and associated molecular pathways, functional assessment of novel nanodomains, to the development of novel tool molecules and new techniques for imaging cyclic AMP compartmentilisation. This editorial aims to summarise these papers within the wider context of cyclic AMP signalling.

## 1. Introduction

Basic research into the ubiquitous cyclic nucleotide second messenger, cyclic 3′,5′-adenosine monophosphate (cyclic AMP), has developed an understanding of how small signalling molecules can mediate the intracellular actions of a wide range of cell surface receptors for hormones, neurotransmitters, and growth factors, as well as sensory stimuli [1,2]. Cyclic AMP is now recognized as a universal regulator of cellular function in a wide variety of organisms, including protozoa, plants, and animals. For example, in this Special Edition, González-Velasco et al., use proteomic and transcriptomic profiling in order to identify early developmentally regulated proteins in Dictyostelium discoideum [3], whereas, in humans, cyclic AMP has been implicated here in the control of the differentiation, proliferation, and apoptosis of stem cells [4], as well as epithelial-to-mesenchymal transition in bronchial epithelial cells, in response to cigarette smoke [5]. In addition, cyclic nucleotide metabolism has been targeted for therapy leading to the development of a series of “block busting” drugs, including beta-blockers (e.g., propranolol), anti-asthma (e.g., salbutamol) and anti-erectile dysfunction (Sildenafil) drugs. Biological processes mediated by cyclic AMP include metabolism, gene regulation, and immune function and its deregulation is associated with many pathologies, including metabolic, psychiatric, cardiovascular, pulmonary, and inflammatory disorders, as well as certain cancers. For example, key studies in this here concentrate on regulatory roles for cyclic AMP in cardiac fibrosis [6], hepatocellular carcinoma [7], restenosis [8], and chronic obstructive pulmonary disorder (COPD) [5]. In addition, Negreiros-Lima et al. described a new role for cyclic AMP in regulating important functions of monocytes/macrophages during the resolution of inflammation. They used a range of animal models and macrophages to demonstrate that cyclic AMP promotes monocyte migration to tissues and macrophage reprogramming to a Th2-like phenotype and the uptake of apoptotic cells [9].

## 2. Control of Cyclic AMP Synthesis

Cyclic AMP is intracellularly synthesised following the activation of GPCRs by physiological stimuli, thereby translating extracellular ligand binding into intracellular signals by virtue of conformational changes in the receptor. These structural effects result in the dissociation of Gα and Gβγ heterotrimeric G-protein subunits in the plasma membrane [10]. It is the stimulatory, Gsα subunit that is specifically involved in the synthesis of cyclic AMP, since it activates adenylate cyclase (AC), thereby catalysing the conversion of ATP into cyclic AMP and pyrophosphate. In contrast, inhibitory Gia subunits block AC activation, thereby limiting cyclic AMP production. An elegant study in this Special Edition explores the role of the gamma subunits in the regulation of localization and activation of Gsα and Giα, while using FRET and mobility measurements [11]. It was shown that the rate of G-protein diffusion depends on the subclass of γ-subunit, and Gsα and Giα exhibited different behaviours [11]. This has relevance for the sequestration of cyclic AMP signalling to discrete sub-cellular compartments, or “nanodomains”, which is now widely viewed as the paradigm for “compartmentalised” cellular signalling in biological systems. For example, air pollution leads to the dysfunction of cells in the cardiovascular system and an increased risk of cardiovascular diseases, including atherosclerosis, hypertension, myocardial infarction, thrombosis, and restricted valve motion [12,13]. Recent data indicate that air pollutants provoke the remodelling of cyclic AMP nanodomain composition, leading to impaired cyclic AMP signalling in model systems [14,15,16], and could feasibly impact on the mobilization of cyclic AMP signalling components in the cardiovascular system. In this regard, AC isoforms are emerging as also contributing to the formation of nanodomains. For example, we see here that the local sympathetic regulation of the potassium current in adult cardiomyocytes and transgenic mice involves the formation of a multi-protein “signalosome” involving AC9 [17]. It has yet to be seen whether diffusion and sequestration of G-protein and AC isoforms are altered during environmental stress and cardiovascular disease; however, the next generation of cyclic AMP modifying drugs are likely to be targeted towards modulating the composition of nano-localised signalling complexes. In this regard, monitoring signalling complex formation in real time might be helped by the design/development of a novel biomolecular fluorescence complementation (BiFC) based high-throughput method of protein-protein interactions in live cells, as described here by Doyle et al. [18]. This method was validated using AC5 as a target in three different cell systems and successfully identified the protein phosphatase 2A (PP2A) catalytic subunit (PPP2CB) and the intracellular trafficking associated protein-NSF (N-ethylmaleimide-sensitive factor) attachment protein alpha (NAPA) as interacting partners [18]. Having established this technique, it will now be interesting to apply it in order to characterize nanodomain remodelling under a range of pathological and environmental conditions.

## 3. Cyclic AMP Effector Proteins–PKA and POPDC Proteins

Activation of effector proteins by cyclic AMP controls many aspects of cellular function, including proliferation [19], differentiation [20], secretion [21], cell spreading [21,22], inflammation [23], contractility [24], and plasma membrane remodelling [24]. To mediate these actions, elevated intracellular cyclic AMP levels can activate a select range of signalling pathways through specific interaction with effector proteins that contain cyclic nucleotide binding domains (CNBs). These include protein kinase A (PKA) [25], exchange protein activated by cyclic AMP (EPACs 1 and 2) [26,27], cyclic AMP responsive ion channels (CICs) [28], and Popeye domain containing proteins (POPDCs) [29]. Signalling from activated PKA is mediated through the phosphorylation of a plethora of intracellular target proteins [30], whereas the activity of EPAC proteins is principally directed towards the stimulation of Rap1 and Rap2 GTPases [26,27] through its inherent guanine nucleotide exchange factor activity. CICs are found within olfactory sensory neurons, brain, kidney, and the heart. Cardiac CICs are called hyperpolarization-activated CICs and they are responsible for maintaining a regular heartbeat [28].

PKA is the best-known cyclic AMP effector and the mechanisms by which this kinase targets protein substrates are described in this special edition. The authors used surface plasmon resonance to perform an in-detail study of the catalytic reaction of the PKA Cα isoform with serine or threonine substrates [31]. Interestingly, magnesium and nucleotides were found allow PKA to target serine and threonine amino acids with similar affinity, however PKA was more specific for serine in their absence. It was also found that threonine-containing substrates have a faster turnover rate and a clinically relevant mutation, F187V, improves the efficiency of PKA towards threonine-containing substrates, which may have relevance for understanding disease processes.

POPDCs are recently discovered membrane bound cyclic AMP receptors that interact with the two-pore domain potassium channel, TREK-1 [32], and Caveolin-3 [33], in order to regulate pacemaker activity. Indeed, a comprehensive review in this Special Edition by the Brand laboratory describes a central role for the POPDC family in organ homeostasis and describes their role in different cellular pathways that are involved in membrane trafficking, cardiac arrhythmia, muscular dystrophy, cell proliferation, migration and invasion [34]. Indeed, deletion of POPDCs produces pronounced cardiac arrhythmia, suggesting that they have a vital role in cardiac function consistent with their targeted expression within cardiomyocytes [29]. Interestingly, the cyclic AMP binding domain of POPDCs displays low sequence homology when compared to other CNBs, suggesting a convergent form of evolution [32]. Future work will no doubt reveal further the importance and mechanisms of action of this new family of cyclic AMP-binding proteins.

## 4. Cyclic AMP Effector Proteins–EPAC Proteins

This Special Edition highlights the important role that EPAC proteins play in cellular physiology. For example, the manuscript, entitled “Epac1 protein: pharmacological modulators, cardiac signalosome and pathophysiology”, by Bouvet et al. [35] presents an overview of the recent advances in pharmacological tools to target EPAC signaling and it illustrates the role of EAPC1 in cardiomyocytes and how dysregulation of EPAC signaling may contribute to the development of cardiac disease. Indeed, the review by Smith et al. [8] details how PKA and EPAC1 suppresses vascular smooth muscle cell proliferation through cytoskeleton remodelling and proliferation-associated gene expression through TEAD transcription factors, highlighting further potential drug targets for the treatment of restenosis after angioplasty, vein graft intimal thickening, and atherosclerosis [8]. These new cyclic AMP-dependent transcription factor pathways are complemented by new findings from the Yarwood lab that demonstrate that EPAC1 and C/EBP and c-Jun transcription factors mediate cyclic AMP-regulated inflammatory gene expression in vascular endothelial cells, which also provides a new drug-able route for the treatment of inflammation that is associated with vascular disease [36].

In terms of potential therapies, work is progressing on the development of selective agonists and agonists towards EPAC1 and EPAC. For example, here, Ahemed et al., review the biophysical properties of the EPAC antagonists CF3F4R and ESI-09 and discuss workable concentration ranges and strategies to overcome inherent limitations of solubility, together with details of proposed mechanisms of action [37]. Work is also progressing in the development of EPAC agonists. To date, the Rehmann group has developed cyclic nucleotide derived activators of EPAC1 and EPAC2, namely D-007 and S-220, respectively [38]. Here, the Rehmann group further describe a newly developed membrane-permeable prodrug, S-223, for selective Epac2 activation in living cells [39]. The first non-cyclic nucleotide activator, I942, was discovered by the Yarwood lab and was shown here to regulate global gene expression patterns associated with vascular inflammation [40]. Subsequently, an I942 analogue, with improved anti-inflammatory properties, was identified [41]. In this Special Edition, the Yarwood group describe the identification of a novel class of Benzofuran Oxoacetic Acid, EPAC1-selective small molecular activators, which are chemically distinct from the I942 class of compounds [42]. This provides chemical flexibility for the future design of EPAC1-selective agonists with anti-inflammatory properties and a new set of tool molecules in order to probe the function of EPAC1 in experimental models.

While crystal structures of EPAC2 exist, in liganded and non-liganded conformations [39,43,44], the development of EPAC1-selective drugs with high potency is hindered by the lack of a crystal structure for EPAC1. Therefore, a significant forward step is presented in this Special Issue. The study entitled “Conformational States of Exchange Protein Directly Activated by cyclic AMP (EPAC1) Revealed by Ensemble Modeling and Integrative Structural Biology” by White et al. [45] uses the biophysical technique, small-angle X-ray scattering (SAXS), in order to demonstrate that EPAC1 has multiple conformations in solution. They describe an autoinhibitory conformational state and a cyclic AMP-induced dimer that could potentially be involved in the functional regulation of EPAC1 [45]. For the first time, they show that the EPAC1 activation proceeds from a compact, inactive state, through a previously unknown cyclic AMP-bound, intermediate-dimer state, to an extended cyclic AMP-bound form that can interact with Rap1 [45]. This complexity will perhaps inform the development of new small-molecule regulators that can target individual transitional states in the EPAC1 activation pathway.

## 5. Compartmentalisation of Cyclic AMP Signalling into Nanodomains

The subcellular localization of cyclic AMP is vitally important in determining the cellular response. Indeed, if a subcellular compartment is rich in cyclic AMP phosphodiesterases (PDEs), it will hydrolyse cyclic AMP to 5′-AMP following synthesis. The cyclic AMP signal will therefore be confined to the vicinity surrounding ACs and, conversely, if PDEs are absent, then the cyclic AMP signal will be more widely distributed and sustained [46]. Cells express a large array of PDE enzymes that degrade cyclic AMP and can control specific cellular processes through targeting and anchoring to specific signalling complexes within the cell. Because of this, the relative abundance of a specific PDE species in cells does not necessarily correspond to their relative importance for cellular functions. Rather, even low-abundance PDEs can be important for the regulation of key cellular processes due to specific targeting to distinct sub-cellular signalling complexes. These “targeted” PDEs can therefore regulate local cyclic AMP levels to control the activity of co-targeted cyclic AMP effectors, including EPAC and PKA. Three papers in the current issue elegantly describe these processes. For example, the paper by Brzezinska and Maurice [47] demonstrated that EPAC1 promotes the formation of the formation of leading-edge protrusions in migrating arterial smooth muscle cells and this is regulated by a localized pool of PDE1C enzymes, but not by the more abundant PDE3 or PDE4 activities that are present in these cells [47]. In addition, Tschaikner et al. [48] describe how a macromolecular, ciliary-localized signaling complex, composed of the orphan GPCR, Gpr161, and type I PKA holoenzymes, is involved in antagonizing Hedgehog signalling, which controls cell fate specification and cell proliferation during embryonic development and in the homeostasis of adult tissue [48]. Moreover, they describe how different PDE isoforms (including PDE1C and PDE4C) control cyclic AMP oscillations in a nanodomain-specific manner in the sensory primary cilium compartment [48]. Finally, in the review conducted by Tibbo and Baillie, the authors describe the functional role of PDE4B in brain [49]. In particular, they describe a key role for a multi-protein complex encompassing Nuclear Distribution Factor-E-like (NDEL1) and its orthologue NDE1, Lissencephaly (LIS1), Disrupted-in-Schizophrenia 1 (DISC1), and PDE4B in the control of neuronal signalling linked to psychiatric disorders, probably through the regulation of the local activity of PKA [49].

It is now clear that the compartmentalisation of cyclic AMP signalling is orchestrated by a range of specialised anchoring proteins, like DISC1, which interact with PDEs and cyclic AMP effector proteins to spatially control the cellular response to increases in intracellular cyclic AMP levels [46,50]. For example, both PKA and EPAC are sequestered to distinct subcellular compartments through the actions of PKA anchoring proteins (AKAPs) [51] and EPAC anchors (e.g., RanBP2, mAKAP, Ezrin, and MAP1a) [52,53,54,55]. The unique targeting of anchoring proteins to individual subcellular compartments, including the cytoskeleton or different organelles, will determine which effector pathway is triggered and, therefore, the nature of the local response following cyclic AMP stimulation. Similarly, PDE4s have also been reported to be recruited to subcellular compartments through interactions with anchoring proteins, including mAKAP, β-arrestin, and RACK1 [53,56,57]. In the case of mAKAP, the coordinated interaction of PKA, EPAC1, and ERK5 leads to local reduction of cyclic AMP levels through the stimulation of AKAP-bound PDE4D3 by PKA, and the release of ERK5-mediated PDE4D3 inhibition through the actions of EPAC1, thereby providing complex regulation of cyclic AMP levels by two coordinated feedback mechanisms [53]. EPAC, PDEs, and members of the AKAP superfamily contribute to the development and progression of COPD and show a profound alteration in their expression patterns in experimental models. The expression of EPAC1 [15,16] and AKAPs 5 and 12 were reduced in airway smooth muscle cells, whereas PDEs 3 and 4 were increased, leading to reduced cyclic AMP levels and airway constriction. The corresponding reduction in EPAC activity resulted in increased cell proliferation and inflammation. In airway epithelial cells, the expression of AKAP9 and the adherens junction marker, E-cadherin, was also found to be decreased [58], leading to a loss in barrier function. These findings point to a central role for cyclic AMP nanodomain complexes in the development of COPD. In the current issue, this work has been expanded upon to implicate AKAPs, including ezrin, Yotiao, and AKAP95, in the mediation of epithelial-to-mesenchymal transition (EMT) induced by TGF-β1 and cigarette smoke in human bronchial epithelial cells and, therefore, plays a role in the development of COPD [5]. The authors show that disruption of PKA anchoring decreases E-cadherin but counteracts TGF-β1-induced collagen1 expression [5]. TGF-β1 alters the expression of ezrin, Yotiao and AKAP95 and knock-out of these AKAPs impacts TGF-β1 inhibits collagen expression and migration of bronchial epithelial cells [5]. They conclude, finally, that AKAPs might underlie the actions of cyclic AMP-elevating drugs, including, fenoterol, rolipram, and cilostamide, to inhibit EMT, and, therefore, might represent new drug targets for the treatment of COPD and, perhaps, the development of lung cancer.

## 6. Imagining Cyclic AMP Signalling into Nanodomains

Real-time imaging techniques to visualize cyclic AMP in cells have greatly enhanced our understanding of the distribution and nature of intracellular cyclic AMP signalling domains, which are organised into multi-protein nanodomains that are regulated in a spatiotemporal manner. Cyclic AMP measurements with FRET-based probes, especially in combination with other powerful techniques, such as scanning ion conductance microscopy (SICM), allowed for narrowing down single cyclic AMP signalling domains to individual T-tubules in cardiomyocytes [59]. Development of targeted sensors first highlighted differences in cytoplasmic and mitochondrial matrix cyclic AMP concentrations [60,61,62], i.e., in compartments that are only few micrometres apart [63]. This was further refined by sensors that were targeted to different compartments of the mitochondria itself [61,62]. Likewise, sensors targeted to the sarcoplasmic reticulum were unable to detect cyclic AMP generated by β2-adrenergic receptors in the T tubules [64], even though these organelles are juxtaposed a few hundred nanometres apart from each other at the dyadic junction of a cardiomyocyte [65]. There are differential responses in cyclic AMP signalling, even between the troponin complex and myosin binding protein C [66], which are located only a few nanometres apart from each other within the sarcomere [67]. Thus, for proper physiological function, it is vital that cyclic AMP signalling occurs in nanodomains. A major advance in this Special Edition is the report of a new software tool, MultiFRET, which, together with a mechanical microscope stage, allows the assessment of multiple cells in the same experiment [68]. This should allow researchers to determine FRET from multiple cells within one specimen and, therefore, very important for producing data from samples where there is a large heterogeneity within the cell population, such as primary cells. Therefore, the researchers examined the FRET responses using multiple probe constructs (pmEpac-1, Epac1-PLN, epac1-cyclic AMPs, AKAP79-CUTie, Epac-SH74) in multiple cell types (adult and neonatal mouse cardiomyocytes and hiPSC cells) [68]. It is envisaged that this approach may be scaled up for high throughput screening in order to identify drugs that target individual nanodomains in a cell population.

## 7. Conclusions

In conclusion, this Special Edition of Cells highlights important new advances in the cyclic AMP signalling field that will give us a greater understanding of the temporal and spatial localization of the cyclic AMP signalling system in cells and how this can impact various disease states, which range from cardiopulmonary disorders to brain disease and cancer. It also describes new experimental tools and imaging techniques that, together, will help to further probe the importance of cyclic AMP nanodomains in health and disease.
